# A comparison of methods for determining the partition coefficient of gadolinium in the myocardium using T1 mapping

**DOI:** 10.1186/1532-429X-13-S1-O81

**Published:** 2011-02-02

**Authors:** Rajesh Janardhanan, Ronny S Jiji, Jeremy Brooks, Frederick H Epstein, Christopher M Kramer, Michael Salerno

**Affiliations:** 1 University of Virginia, Charlottesville, VA, USA

## Introduction

Late gadolinium enhancement can evaluate focal, but not diffuse myocardial fibrosis. T1-mapping techniques can quantify fibrosis by calculating the partition coefficient (λ) of gadolinium (Gd). One method (CI) calculates λ at equilibrium following a continuous infusion of Gd, while the other, early post-contrast method (EPC), determines λ from multiple post-contrast time points after Gd injection. The Modified Look-Locker Inversion Recovery (MOLLI) technique accurately performs T1 mapping, but may be limited in practice due to the long breathhold required. A shortened-MOLLI technique (Sh-MOLLI) has been described, which only allows a single heart beat for magnetization recovery.

## Objectives

To (i) compare T1 values determined with reduced breathhold MOLLI schemes to the standard MOLLI technique, and (ii) to directly compare λ determined by the CI and EPC methods.

## Methods

T1 mapping was performed in 10 healthy volunteers (age 34±11) on a Siemens 1.5T Avanto using 3 MOLLI schemes: (i) Standard MOLLI (ii) 3-5 MOLLI (11 heart beats, 2 inversions, 3 recovery beats, 8 images), (iii) 2-2-4 MOLLI (12 heart beats, 3 inversions, 2 recovery beats, 8 images). Sequence Parameters included: TE/TR/FA 1.1 ms/2.5ms/35°, FOV= 340 x 260, resolution 1.8mm x 1.8mm, thickness 8mm. T1 was determined pre-contrast and every 5 minutes following injection of 0.1mmol/kg Gd-DTPA. At 15 minutes a continuous infusion of 0.001 mmol/kg Gd was administered until equilibrium was achieved. λ was determined using the CI and EPC methods. T1 maps were calculated and manually segmented using an in-house MATLAB program.

## Results

**Figure 1:**
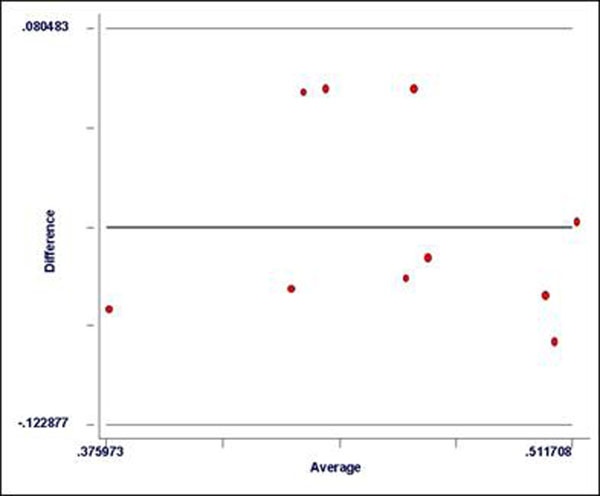


The standard MOLLI and the 3-5 MOLLI sequences provided comparable T1 values for myocardium and blood both pre- and post-contrast, whereas the 2-2-4 MOLLI sequence had a bias towards lower T1 values pre-contrast (see table 1). There was good agreement between the partition co-efficient of gadolinium for the CI method and for the EPC method (see Figure 1).

**Table 1:** Mean T1 values and partition co-efficients observed

Mean Values	Standard MOLLI	3-5 MOLLI	2-2-4 MOLLI	P-value
**T1 of blood pre-contrast (msec)**	1478±86	1480±100	1390±91	0.06
**T1 of myocardium pre-contrast (msec)**	972±27	953±38	919±84	0.11
**T1 of blood post-contrast (msec)**	488±36	487±43	484±44	0.97
**T1 of myocardium post-contrast (msec)**	601±37	597±37	600±30	0.96
**Partition Co-efficients (CI-based method)**	0.47±0.04	0.45±0.05	0.45±0.03	0.39
**Partition Co-efficients (EPC-based method)**	0.49±0.05	0.47±0.05	0.45±0.04	0.20

## Conclusions

The 3-5 MOLLI sequence produces reliable T1 maps in a short clinically-applicable breathhold and is comparable to the standard approach. The 2-2-4 MOLLI sequence had a bias towards lower T1 values pre-contrast, possibly due to insufficient T1 relaxation between inversion pulses. The λ obtained by either the CI or EPC methods were similar. Using the EPC method, T1 mapping can quantify diffuse myocardial fibrosis without requiring a long equilibrium phase.

